# Transcriptional Rewiring, Adaptation, and the Role of Gene Duplication in the Metabolism of Ethanol of Saccharomyces cerevisiae

**DOI:** 10.1128/mSystems.00416-20

**Published:** 2020-08-11

**Authors:** Beatriz Sabater-Muñoz, Florian Mattenberger, Mario A. Fares, Christina Toft

**Affiliations:** a Department of Abiotic Stress, Instituto de Biología Molecular y Celular de Plantas (IBMCP), Consejo Superior de Investigaciones Científicas-Universidad Politécnica (CSIC-UPV), Valencia, Spain; b Smurfit Institute of Genetics, Department of Genetics, University of Dublin, Trinity College, Dublin, Ireland; c Program for Systems Biology of Molecular Interactions and Regulation, Institute for Integrative Systems Biology (I^2^SysBio, CSIC-UV), Valencia, Spain; Oxford Nanopore Technologies

**Keywords:** RNAseq, adaptive laboratory experimental evolution, clonal populations, transcriptional divergence, ethanol stress

## Abstract

Ethanol is the main by-product of yeast sugar fermentation that affects microbial growth parameters, being considered a dual molecule, a nutrient and a stressor. Previous works demonstrated that the budding yeast arose after an ancient hybridization process resulted in a tier of duplicated genes within its genome, many of them with implications in this ethanol “produce-accumulate-consume” strategy. The evolutionary link between ethanol production, consumption, and tolerance versus ploidy and stability of the hybrids is an ongoing debatable issue. The implication of ancestral duplicates in this metabolic rewiring, and how these duplicates differ transcriptionally, remains unsolved. Here, we study the transcriptomic adaptive signatures to ethanol as a nonfermentative carbon source to sustain clonal yeast growth by experimental evolution, emphasizing the role of duplicated genes in the adaptive process. As expected, ethanol was able to sustain growth but at a lower rate than glucose. Our results demonstrate that in asexual populations a complete transcriptomic rewiring was produced, strikingly by downregulation of duplicated genes, mainly whole-genome duplicates, whereas small-scale duplicates exhibited significant transcriptional divergence between copies. Overall, this study contributes to the understanding of evolution after gene duplication, linking transcriptional divergence with duplicates’ fate in a multigene trait as ethanol tolerance.

**IMPORTANCE** Gene duplication events have been related with increasing biological complexity through the tree of life, but also with illnesses, including cancer. Early evolutionary theories indicated that duplicated genes could explore alternative functions due to relaxation of selective constraints in one of the copies, as the other remains as ancestral-function backup. In unicellular eukaryotes like yeasts, it has been demonstrated that the fate and persistence of duplicates depend on duplication mechanism (whole-genome or small-scale events), shaping their actual genomes. Although it has been shown that small-scale duplicates tend to innovate and whole-genome duplicates specialize in ancestral functions, the implication of duplicates’ transcriptional plasticity and transcriptional divergence on environmental and metabolic responses remains largely obscure. Here, by experimental adaptive evolution, we show that Saccharomyces cerevisiae is able to respond to metabolic stress (ethanol as nonfermentative carbon source) due to the persistence of duplicated genes. These duplicates respond by transcriptional rewiring, depending on their transcriptional background. Our results shed light on the mechanisms that determine the role of duplicates, and on their evolvability.

## Abstract (precis)

Gene duplication events have been related with increasing biological complexity through the tree of life, but also with illnesses, including cancer. Early evolutionary theories indicated that duplicated genes could explore alternative functions due to relaxation of selective constraints in one of the copies, as the other remains as ancestral-function backup. In unicellular eukaryotes like yeasts, it has been demonstrated that the fate and persistence of duplicates depend on duplication mechanism (whole-genome or small-scale events), shaping their actual genomes. Although it has been shown that small-scale duplicates tend to innovate and whole-genome duplicates specialize in ancestral functions, the implication of duplicates’ transcriptional plasticity and transcriptional divergence on environmental and metabolic responses remains largely obscure. Here, by experimental adaptive evolution, we show that Saccharomyces cerevisiae is able to respond to metabolic stress (ethanol as nonfermentative carbon source) due to the persistence of duplicated genes. These duplicates respond by transcriptional rewiring, depending on their transcriptional background. Our results shed light on the mechanisms that determine the role of duplicates, and on their evolvability.

## INTRODUCTION

Sensing and responding to the environment are central parts of metabolism of almost all unicellular organisms. During evolution, some budding yeasts (Saccharomycotina) faced a new source of carbon (sugars) in a new niche (nectar or fruits from the recently emerged [100 million years ago {MYA}] Angiosperms), a fact that has been postulated at the origin of fermentative metabolism with ethanol as main end product. Behind this biological innovation has been unveiled gene duplication at two scales, whole-genome (WGD) and small-scale (SSD) duplication, and genome shrinkage after it, as evolutionarily driving genomic changes (reviewed in references 1 to 3). The baker’s yeast Saccharomyces cerevisiae is one of the most biotechnologically important species, being able to tolerate higher ethanol levels during fermentation than any other microbe (4–6).

Under sugar scarcity, yeast can switch from fermentative to respiratory metabolism using ethanol and glycerol as nonfermentative carbon sources to support growth (7, 8). This ethanol “make-accumulate-consume” strategy (Crabtree effect) has been partially linked to the yeast evolutionary origin history. However, ethanol in particular endangers the yeast metabolic activity, survival, cell morphology, growth ability, and biomass production. Ethanol also exhibits a general cell toxicity that yeasts used to control competitors’ growth. This duality (nutrient and stressor) generates great concerns in the biotechnological industries (by its applications) and in the scientific community (by its molecular basis), highlighting the importance of systems biology studies (reviewed in references 9 to 12).

Experimental evolution, in particular with Escherichia coli and S. cerevisiae, has been of unprecedented relevance to unveil evolutionary pathways underlying the origin of adaptations, including as examples the adaptation of E. coli to citrate in the known Lenski evolution experiment (13–17) and heat stress, nutrient limitations, antibiotic treatment, or tolerance to glycerol in S. cerevisiae (18–24). Its use to understand the adaptation to ethanol has been addressed in only a few studies, while using ethanol as additional carbon source (4, 5, 25). Indeed, only one work revealed the genomic dynamics including point mutations, copy number variation (gene duplication), ploidy changes, and clonal interference mix in a complex evolutionary pathway that increases tolerance to ethanol (4). Nonetheless, the transcriptional rewiring occurring during this response to ethanol and its importance in comparison with the contribution of genomic changes have not been explored. Indeed, the implication in ethanol response and adaptation of duplicates, from a transcriptional perspective, have been only marginally explored recently by our group (19, 23). The interplay between duplicates and transcriptional rewiring remains unknown. It also remains elusive whether and how S. cerevisiae could optimize the use of ethanol as nonfermentative carbon source.

In this study, we undertake the challenge of elucidating the role of transcriptional rewiring to the response and adaptation to ethanol (as sole carbon source) in S. cerevisiae and revealing the link between gene duplication and ethanol usage. As already mentioned, previous studies revealed an unprecedented complexity in the genomic dynamics underlying adaptation to ethanol but, however, did not address the implication of transcriptional reprogramming of the ancestral duplicates (4, 5, 25). Here, we evolved clonal populations of S. cerevisiae using glucose as carbon source and challenged them to use ethanol as sole carbon source in short and long (ethanol adaptive laboratory evolution) responses. We reveal the transcriptional reprogramming basis and the interplay of this with gene duplication in the response and adaptation to ethanol.

## RESULTS

### Phenotypic changes of S. cerevisiae in response and adaptation to ethanol.

**FIG 1:**
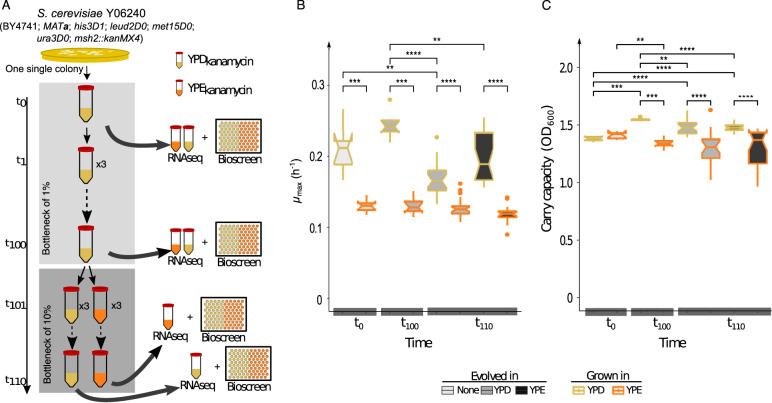
Experimental adaptive laboratory evolution scheme and phenotypic characterization in Saccharomyces cerevisiae Y06240. (A) Experimental layout. From a single S. cerevisiae Y06240 (*MAT***a**; *his3D1; leud2D0; met15D0; ura3D0; msh2*::*kanMX4*) colony, we derived a population in liquid YPD medium (called population t_0_). This population was split into 3 replicates and evolved in YPD for 100 passages (approximately 660 generations) by daily transferring 1% (0.5 ml) to a new tube (50 ml) with fresh YPD medium (4.5 ml) (we called this population t_100_). After passage 100, we started the adaptive evolution, by splitting up the evolutionary experiment into two: one continuing to evolve in YPD (with 2% glucose as carbon source) and the other replacing the glucose with 3% ethanol (medium YPE). Populations were evolved for 10 passages with a daily 10% bottleneck (approximately 33 generations; we called this population t_110_). In the experimental scheme, the points at which phenotypic characterization and transcriptome changes (RNAseq) were carried out are indicated. (B) Phenotypic characterization was performed by characterization of population growth curves. Maximum growth rate (h^−1^) of each population was determined at each control time point (t_0_, t_100_, and t_110_) in their evolving medium (YPD or YPE) and in the challenge medium (YPE or YPD). (C) “Carry capacity” of each population (OD_600_) was also determined for each population and each control time point in their evolving medium and in the challenge one. Significant differences of each growth parameter are indicated as *, **, ***, and ****, when the probabilities are *P* < 0.05, *P* < 0.005, *P* < 10^−3^, and *P* < 10^−4^, respectively, using a Wilcoxon rank test.

At time points t_0_, t_100_, and t_110_, we characterized growth parameters of S. cerevisiae populations in the standard medium (yeast extract-peptone-dextrose [YPD]) and stressful medium (yeast extract-peptone-ethanol [YPE]), using optical density measurements (Fig. 1A). The mean maximum growth rate (μ_max_) was significantly lower at time t_0_ in YPE (μ_max_ ± standard deviation of the mean [SDm] = 0.1303 ± 0.0093 h^−1^) than in YPD (μ_max_ ± SDm = 0.2096 ± 0.0343 h^−1^; Wilcoxon rate test, *P* = 5.8 × 10^−4^). This difference between growth rates was also observed in all the evolved lines at all time points (Fig. 1B; see also Fig. S1 in the supplemental material). Diversifying the population for approximately 660 generations (t_100_) increased the growth rate in YPD for all lines. Furthermore, the populations also increase their carry capacity after diversification (Fig. 1C; Fig. S2). However, only one of the lines increased its growth rate in YPE; the other two retained a similar growth rate as the ancestral population (Fig. S1 and S2). All lines, except one, at time t_110_ reduced the growth rate after just 33 generations. The populations evolved in YPE and when challenged to grow in YPD showed a higher growth rate than the control population evolved in YPD. This difference comes from the growth rate recovery of one of the evolved populations in YPE. The rest of the populations in t_100_ perform similarly. Overall, the evolved population in YPE reduced their growth rate in YPE compared to the evolved populations in YPD but increased their carry capacity (Fig. S1 and S2).

10.1128/mSystems.00416-20.1 FIG S1 Maximum growth rate as phenotypic characterization of populations through experimental evolution. Phenotypic characterization was performed by characterization of population growth curves. “Maximum growth rate” (h^−1^) of each population, split out into the different lines, was determined at each control time point (t_0_, t_100_, and t_110_) in their evolving medium (YPD or YPE) and in the challenge medium (YPE or YPD). Significant differences of each growth parameter are indicated as *, **, and ***, when the probabilities are *P* < 0.05, *P* < 0.005, and *P* < 10^−3^, respectively, using a Wilcoxon rank test. Only biologically meaningful comparisons were added to the figure. Download FIG S1, EPS file, 0.1 MB. Copyright © 2020 Sabater-Muñoz et al. 2020 Sabater-Muñoz et al. This content is distributed under the terms of the Creative Commons Attribution 4.0 International license.

10.1128/mSystems.00416-20.2 FIG S2 Evolution of carry capacity of populations under ethanol stress. Phenotypic characterization was performed by characterization of population growth curves. “Carry capacity” (OD_600_) of each population, split out into the different lines, was determined at each control time point (t_0_, t_100_, and t_110_) in their evolving medium (YPD or YPE) and in the challenge medium (YPE or YPD). Significant differences of each growth parameter are indicated as *, **, and ***, when the probabilities are *P* < 0.05, *P* < 0.005, and *P* < 10^−3^, respectively, using a Wilcoxon rank test. Only biologically meaningful comparisons were added to the figure. Download FIG S2, EPS file, 0.1 MB. Copyright © 2020 Sabater-Muñoz et al. 2020 Sabater-Muñoz et al. This content is distributed under the terms of the Creative Commons Attribution 4.0 International license.

### Up- and downregulation in response and adaptation to ethanol.

Transcriptome sequencing (RNAseq) was conducted in populations t_0_, t_100_, and t_110_, in YPD and/or challenged with ethanol (YPE) (Fig. 1A). The exposure to ethanol led to the upregulation (fold change [FC] in the expression of the genes >25%, false-discovery rate [FDR] <0.005) of 833 and 1,389 genes compared to the same population grown in YPD for t_0_ and t_100_, respectively. Of the 833 genes upregulated in t_0_, 557 (66.9%) were also upregulated in t_100_ (Fig. S3A). Adaptation to ethanol stress for 10 passages led to the upregulation of 1,694 genes, of which 437 were also upregulated in t_0_ and t_100_ (we call these core upregulated genes) (Fig. S3A). The exposure to ethanol led to the downregulation of 751 and 940 genes compared to the same population grown in YPD for t_0_ and t_100_, respectively. Of the 751 downregulated genes in t_0_, 326 (43.4%) were also downregulated in t_100_ (Fig. S3B). The adaptation to ethanol stress led to the downregulation of 1,391 genes, of which 222 were also downregulated in t_0_ and t_100_.

10.1128/mSystems.00416-20.3 FIG S3 Distribution of transcriptomic profiles under ethanol stress. Venn diagrams of up- and downregulated genes in YPE compared to YPD. Download FIG S3, EPS file, 0.2 MB. Copyright © 2020 Sabater-Muñoz et al. 2020 Sabater-Muñoz et al. This content is distributed under the terms of the Creative Commons Attribution 4.0 International license.

### Low overlap in the transcriptomic response and adaptation to ethanol.

The number of upregulated genes among the populations t_0_ and t_100_ (67% of t_0_ upregulated genes are also upregulated in t_100_) was high for the t_0_ population, but t_100_ showed twice as many upregulated genes as t_0_, perhaps indicating that experimental evolution in YPD for 100 passages has involved significant polymorphism in the transcriptomic reprogramming of cells in this population. Only 437 genes were core upregulated genes in all three populations. Populations t_110_, the populations derived from t_100_ and evolved for 10 days in ethanol, showed an overlap of only 883 upregulated genes with their parental t_100_ populations despite the low number of passages separating them (Fig. S3A).

**FIG 2:**
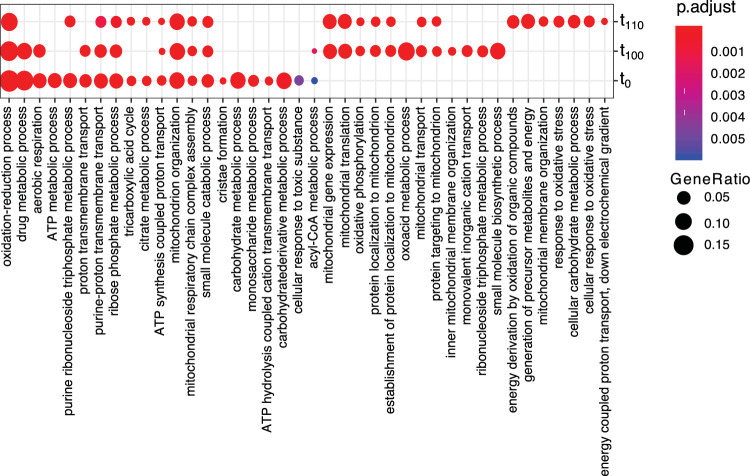
Biological processes enriched due to the use of 3% ethanol as the sole carbon source. Enrichment analysis of functional categories (biological process) for upregulated genes in YPE compared to YPD, at the three time points (t_0_, t_100_, and t_110_), was performed with clusterProfiler.

To determine whether the functions affected by the transcriptomic responses have changed among populations, we performed an analysis of Gene Ontology (GO) terms of the set of upregulated genes. Populations at t_0_ and t_100_ exhibited enrichment for upregulated genes (*P* < 0.01) in similar functional categories, affecting mainly the “oxidation-reduction process,” “drug metabolic process,” “aerobic respiration,” “proton transmembrane transport,” “mitochondrion organization,” “small-molecule catabolic process,” “oxidoreductase activity,” “cofactor binding,” and “proton transmembrane transporter activity” (Fig. 2). The analysis, on the other hand, of GO term enrichment for upregulated genes in t_110_ population led to a somewhat different result. There was some overlap of enriched GO terms from t_0_ and t_110_, but more importantly, a number of GO term enrichments were specific for t_110_. They include terms “energy derivation by oxidation of organic compounds,” “response to oxidative stress,” and “cellular response to oxidative stress and response to inorganic substances” (Fig. 2).

The GO term analysis for downregulated genes showed even less overlap between the three populations (Fig. S4). Population t_0_ had enrichment in “cytoplasmic translation and ribosome biogenesis,” which was also enriched at population t_110_. Furthermore, population t_110_ had “ncRNA processing and methylation” enriched for downregulated genes. In contrast, population t_100_ had only a few GO terms enriched, including “carbohydrate transport” and “nucleic acid phosphodiester bond hydrolysis,” with none of them overlapping the other two populations.

10.1128/mSystems.00416-20.4 FIG S4 Biological processes enriched for downregulated genes, due to the use of 3% ethanol as the sole carbon source. Enrichment analysis of functional categories (biological process) for downregulated genes in YPE compared to YPD, at the three time points (t_0_, t_100_, and t_110_), was performed with clusterProfiler. Download FIG S4, EPS file, 0.1 MB. Copyright © 2020 Sabater-Muñoz et al. 2020 Sabater-Muñoz et al. This content is distributed under the terms of the Creative Commons Attribution 4.0 International license.

### Duplicated genes encode rapid responses and adaptations to ethanol.

Since duplicated genes are involved in the origin of new functions (3, 26–30), we sought to investigate if the response to ethanol, as the sole carbon source, was mainly driven by duplicates, differentiating between WGDs (27) and SSDs (28) in our analyses.

**FIG 3:**
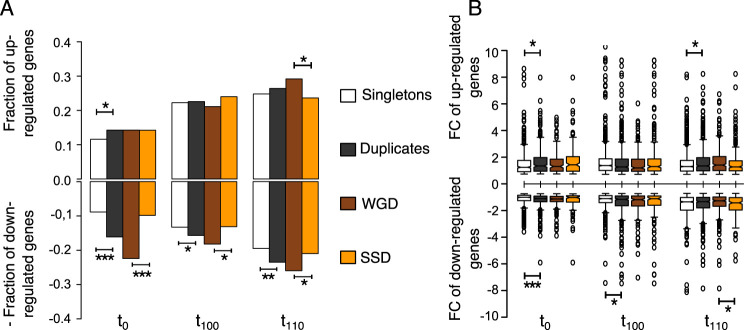
Genes responding transcriptionally to glucose replacement by ethanol, as carbon source, after adaptive evolution. (A) Proportion of responding genes (showing transcriptional divergence [TD]) distributed in four categories (singletons, duplicates, WGDs, and SSDs). Upregulated genes are on the positive part of the *y* axis, whereas downregulated genes are on the negative part of the axis (after being made negative for representation purposes). Fisher’s exact test has been used to test if the observed fractions of TD genes are significantly different from those expected. (B) Expression difference (log fold change) in the two media, YPD and YPE. A Wilcoxon rank test has been used to test the difference in expression levels of sets of genes. Significant differences are indicated as *, **, and ***, when the probabilities are *P* < 0.05, *P* < 0.005, and *P* < 10^−3^, respectively.

Population t_0_ exhibited 312 duplicate (14.2%) and 524 singleton (11.6%) genes out of the 833 upregulated genes. The proportion of upregulated duplicates was higher than that of singletons (Fisher’s exact test: odds ratio *F *= 1.22, *P* = 0.0071) (Fig. 3A). We also observed a higher expression fold change (FC) difference in duplicates (median FC = 1.343) than in singletons (median FC = 1.244) (Wilcoxon rank test: *P* = 0.0215) (Fig. 3B). We found no difference in the response of duplicates when analyzing their origin (312 = 156 WGDs + 156 SSDs) (Fisher’s exact test: odds ratio *F *= 1.002, *P* = 1). Likewise, no difference was observed in the expression fold change between WGDs (median FC = 2.92) and SSDs (median FC = 3.13) (Wilcoxon rank test: *P* = 0.65).

Population t_100_ showed no difference in the response to ethanol between upregulated duplicates (494) and singletons (1,000) (Fisher’s exact test: odds ratio *F *= 1.01, *P* = 0.807). Remarkably, while the proportion of duplicates that were upregulated increased 58% after 100 passages of evolution, the proportion of singletons increased 92% in comparison with the parental population t_0_ (Fig. 3A). No difference in expression fold change was observed between duplicates and singletons, nor between WGDs and SSDs.

Population t_110_ exhibited 578 upregulated duplicates and 1,116 upregulated singletons, not being significantly different (Fisher’s exact test: odds ratio *F *= 1.06, *P* = 0.285). Interestingly, upregulated duplicates (median FC = 1.346) saw a higher expression fold change than singletons (median FC = 1.298) (Wilcoxon rank test: *P* = 0.0288). We found significantly more WGDs (319) responding to ethanol than SSDs (259) (Fisher’s exact test: odds ratio *F *= 1.23, *P* = 0.0248), but no difference of expression fold change between the two (WGDs: median FC = 1.408; SSDs: median FC = 1.274; Wilcoxon rank test: *P* = 0.0586).

The response of duplicates to ethanol was even more apparent when looking at downregulated genes. Population t_0_ showed a larger proportion of the duplicates (353) being downregulated than singletons (398) (Fisher’s exact test: odds ratio *F *= 1.82, *P* = 2.33 × 10^−14^). Not only were there more ethanol-responding duplicates, but the response was also higher (duplicates: median FC = −1.103; singletons: median FC = −1.004; Wilcoxon rank test: *P* = 3.75 × 10^−4^). Most of this response was coming from WGDs (WGDs: 245; SSDs: 108; Fisher’s exact test: odds ratio *F *= 2.27, *P* = 8.31 × 10^−12^). No difference in the expression fold change was found between the two types of duplicates (WGDs: median FC = −1.130; SSDs: median FC = −1.019; Wilcoxon rank test: *P* = 0.1804).

Population t_100_ showed similar results as population t_0_: 343 of the downregulated genes were duplicates and 597 were singletons (Fisher’s exact test: odds ratio *F *= 1.18, *P* = 0.024). The expression fold change was also higher in duplicates (median FC = −1.158) than in singletons (median FC = −1.108) (Wilcoxon rank test: *P* = 0.0174). More WGDs (199) were responding to ethanol stress than SSDs (144) (Fisher exact test: odds ratio *F *= 1.39, *P* = 6.28 × 10^−3^), but no difference in the expression fold change was observed (WGDs: median FC = −1.186; SSDs: median FC = −1.113; Wilcoxon rank test: *P* = 0.484).

Population t_110_ had no difference in duplicated genes (514) being downregulated compared to singletons (877) (Fisher exact test: odds ratio *F *= 1.20, *P* = 2.71 × 10^−3^). However, duplicated genes had a higher expression fold change than singletons (duplicates: median FC = −1.332; singletons: median FC = −1.349; Wilcoxon rank test: *P* = 0.338). Interestingly, WGDs (284) were more abundant than SSDs (230) (Fisher’s exact test: odds ratio *F *= 1.23, *P* = 0.031), but SSDs (median FC = −1.421) showed a higher expression fold change than WGDs (median FC = −1.271) (Wilcoxon rank test: *P* = 0.0266). The core gene of the downregulated genes consisted of more duplicates (4.3%) than singletons (2.84%) (Fisher’s exact test: odds ratio *F *= 2.36, *P* = 1.27 × 10^−4^), with WGDs as the most affected duplicates (WGD 6.02%; SSD 2.5%: Fisher’s exact test: odds ratio *F *= 2.24, *P* = 1.63 × 10^−8^).

### Transcriptional divergence between duplicates gene copies is linked to the response and adaptation to ethanol in S. cerevisiae.

**FIG 4:**
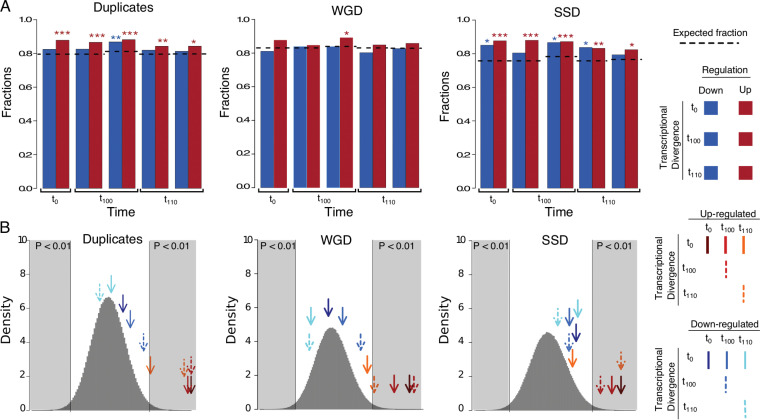
Proportion and mean transcriptional divergence of transcriptionally responding duplicates. (A) The proportion of TD duplicates of transcriptionally responding genes, along with the expected proportions, marked with dashed lines. Significant differences in the observed number of TDs compared to the expected number are indicated as *, **, and ***, when the probabilities are *P* < 0.05, *P* < 0.005, and *P* < 10^−3^, respectively, using a binomial test. (B) Mean transcriptional divergence levels between duplicated genes within each of the categories (marked with arrows) are mapped onto a normal distribution build by random sampling, without replacement, of the same size from the corresponding gene pools. The gray blocks are indicating the significant part of the distribution (*P* < 0.01).

If duplicates were linked to the response and adaptation of S. cerevisiae to ethanol, then we should expect the transcriptional divergence (TD) between gene copies of a duplicate to be correlated with its transcriptional patterns in ethanol. We identified those duplicated genes that exhibited a fold change expression difference between their gene copies of more than 25%. Of the 1,090 duplicated gene pairs (analysis contained both copies), 867 showed transcriptional divergence between gene copies in YPD. In the populations t_0_, 274 of the 312 upregulated duplicates in ethanol belonged to duplicates with evidence of TD, a proportion greater than expected by chance (binomial test: *P* = 1.846 × 10^−4^) (Fig. 4A). The mean expression fold change (in logarithmic scale) of the 274 duplicates was 1.59. We compared this mean to a null distribution of means built by sampling 274 duplicates from the population of the 867 duplicates with evidence of expression divergence (Fig. 4B). The mean fold change of these duplicates was greater than expected by chance (*P* = 3.0 × 10^−6^). The fold change of the gene copy with the highest expression in ethanol divided by that of the least expressed gene copy is also correlated with the expression fold change of the duplicate (Pearson correlation: *r *= 0.66, *P* < 2.2 × 10^−12^). Importantly, among the most highly divergent and upregulated duplicates, we identified the plasma membrane H^+^-ATPase (*PMA2*), translational elongation factor (*HEF3*), plasma membrane permeases (*GIT1* and *SEO1*), and a gene involved in the metabolism under respiratory conditions (*RGI2*), among others (Table S1).

10.1128/mSystems.00416-20.7 TABLE S1 Divergent levels and transcriptional difference between YPE and YPD of duplicated genes. Duplicated genes are divided into whole-genome duplicates (Ohn) and small-scale duplicates (par). t0E.t0D is the log fold change of the expression difference between YPE and YPD, where d_t0E.t0D indicates if the gene has been upregulated (1) or downregulated (−1) or if no difference has been observed (0). Download Table S1, CSV file, 0.1 MB. Copyright © 2020 Sabater-Muñoz et al. 2020 Sabater-Muñoz et al. This content is distributed under the terms of the Creative Commons Attribution 4.0 International license.

In the t_100_ population 426 duplicates of the 492 showed evidence of upregulation and belonged to the set of duplicates with evidence of expression divergence, a proportion greater than expected by chance (binomial test: *P* = 6.81 × 10^−5^). Like in the t_0_ population, upregulated duplicates exhibited greater mean expression divergence between gene copies than expected by chance (mean = 1.45, *P* = 1.05 × 10^−3^) (Fig. 4B). The phenotypic plasticity (expression fold change of duplicates in ethanol compared to YPD) was correlated with the expression divergence between the gene copies (Pearson correlation: *r *= 0.58, *P* < 2.2 × 10^−16^).

Population t_110_ also presented enrichment of upregulated duplicates for duplicates with evidence of expression divergence, with 485 out of the 576 upregulated duplicates exhibiting expression divergence (binomial test: *P* = 5.22 × 10^−3^). Interestingly, upregulated duplicated genes in t_110_ do not show higher transcriptional divergence than expected when calculated from the t_0_ population (mean = 1.29, *P* = 0.150) but do show such when calculated from the t_110_ population (mean = 1.38, *P* = 0.00120), indicating that transcriptional divergence and upregulation are highly dependent on the current transcriptional background. Similar to the t_0_ and t_100_ populations, the population of t_110_ shows correlation between phenotypic plasticity and expression divergence of duplicated genes (Pearson correlation: *r *= 0.5, *P* < 2.2e−16).

In contrast to this pattern for the upregulated genes, we see no correlation between TD and downregulated genes, at any of the time points (Fig. 4). The only correlation that is also present for the downregulated genes is the phenotypic plasticity in YPD and YPE (Pearson correlations: t_0_, *r* = 0.68, *P* < 2.2e−16; t_100_, *r* = 0.67, *P* < 2.2e−16; t_110_, *r* = 0.61, *P* < 2.2e−16).

**FIG 5:**
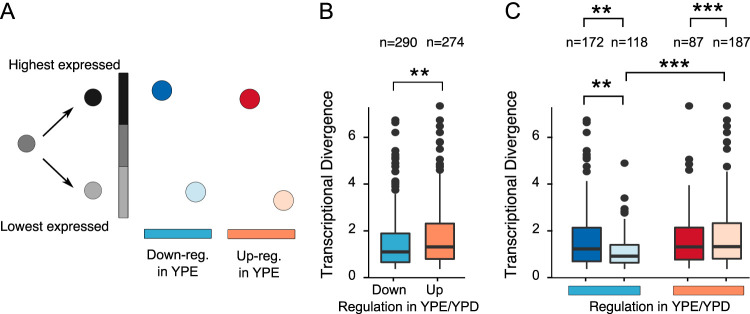
Transcriptional divergence of transcriptionally responding duplicates. (A) Transcriptionally divergent (TD) duplicate characterization. TD duplicates are classified according to their sign of expression (high expression shown in dark colors and low expression shown in light colors), as the responding gene can be either the gene with high expression or the gene with low expression of the duplicated pair. Blue and red indicate the downregulated and upregulated pairs in YPE, respectively. (B) Comparison of TDs of up- and downregulated genes at t_0_. (C) Comparison of TD pairs at t_0_, differentiating each up-expressed gene into high- and low-expression gene categories as indicated in the scheme depicted in panel A. Significant differences are indicated as *, **, and ***, when the probabilities are *P* < 0.05, *P* < 0.005, and *P* < 10^−3^, respectively. A Wilcoxon rank test was used for testing the significance between TDs of the different categories, whereas a binomial test was used for testing the number of TDs in the different categories.

**FIG 6:**
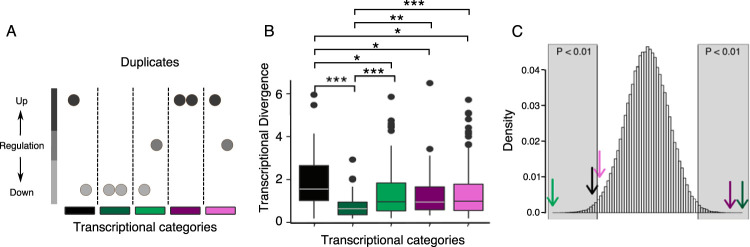
Characterization of TD transcriptionally responding duplicated pairs. (A) TD duplicate categorization scheme (five categories): black, one gene copy is upregulated and the other is downregulated; dark green, both duplicated genes are downregulated; light green, one duplicate is downregulated and the other is unaltered; purple, both duplicates are upregulated; violet, one duplicated gene is upregulated and the other is unaltered. (B) Comparison of TDs of the five categories described. A Wilcoxon rank test was used to determine significant differences indicated as *, **, and ***, when the probabilities are *P* < 0.05, *P* < 0.005, and *P* < 10^−3^, respectively. (C) Mean number of genes within each of the TD categories (marked with arrows with the coloring code described for panel A), mapped onto a normal distribution build by random sampling, without replacement, of the same size from the corresponding gene pools. Gray blocks over the normal distribution indicate the significant part of the distribution (*P* < 0.01).

Understanding why up- and downregulated genes show different patterns with respect to TD, we first checked the overall TD of up- and downregulated genes (Fig. 5B). Downregulated duplicates had significantly lower TD than upregulated genes (*P* = 0.00244). The duplicated genes we are looking at are TD, which means we have one copy with a lower expression than the other (Fig. 5A). Dividing up- and downregulated genes into low and high transcriptional diverged copy (TDC), we observe, as expected, higher TDC in downregulated genes (binomial test: *P* = 0.0018) and lower TDC in upregulated genes (binomial test: *P* = 1.473 × 10^−9^) (Fig. 5C). All groups showed similar TD except for downregulated and low TDC, which had the lowest TD of all groups (Fig. 5C). Looking at GO enrichments of the four categories of Fig. 5C, no overlap is observed between the upregulated and downregulated categories (Fig. S5). Upregulated (highest transcribed copy) genes were enriched for “drug metabolic process,” “energy derivation by oxidation of organic compounds,” and “small molecule metabolic process,” and downregulated duplicates were enriched for “cytoplasmic transition” and different ribosome processes. To determine if the behavior of a gene has influence on the response of the other duplicated copies, we further divided the groups into categories of the two copies having the same regulation profile, the two copies having different regulation profiles, or only one of the copies showing up- or downregulation in ethanol (Fig. 6). Interestingly, we observed more duplicated copies which were both downregulated than expected (*P* < 10^−12^), but this group also had the lowest TD. Inspecting the function of these genes, we see that a majority (48 out of the 60 genes) are ribosomal proteins. As would be expected, a lot of overlap of enriched GOs was observed between the different up- and downregulated categories. In the case of both duplicates being upregulated, we saw an enrichment for “carbohydrate metabolic process” and “oxidative phosphorylation.” Furthermore, these categories are the only ones which observe an enrichment of a pathway, namely, “superpathway of TCA cycle and glyoxylate cycle.” For the discordant duplicates, enrichment categories included “glucose 6-phosphate metabolic process,” “NADP metabolic process,” and “oxidoreduction coenzyme metabolic process” (Fig. S5).

10.1128/mSystems.00416-20.5 FIG S5 Biological processes enriched for transcriptionally divergent duplicated genes. Enrichment analysis of functional categories (biological process) for down- and upregulated transcriptionally divergent duplicated genes in YPE compared to YPD was performed with clusterProfiler. Download FIG S5, EPS file, 0.2 MB. Copyright © 2020 Sabater-Muñoz et al. 2020 Sabater-Muñoz et al. This content is distributed under the terms of the Creative Commons Attribution 4.0 International license.

**FIG 7:**
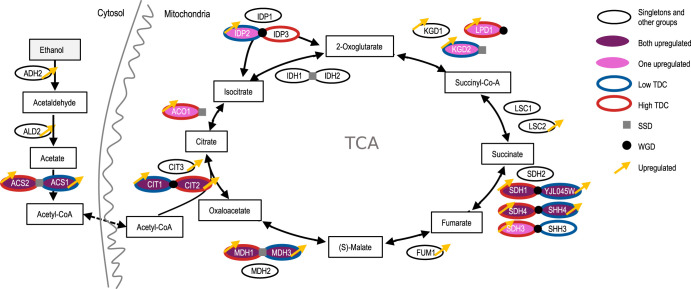
Pathway of nonfermentative C_2_ metabolism in S. cerevisiae Y06240, from ethanol to TCA. Only ethanol degradation and the TCA pathway have been shown. Pathway information was taken from the KEGG pathway database. The proteins are colored after the duplicate categories set out in Fig. 5 and 6, with indication of duplicate origin (SSD or WGD) and transcription-diverged copy level (TDC, in blue or red for low or high, respectively) or upregulation (yellow arrow).

Changing the carbon source from glucose to ethanol implies that the yeast goes from fermentation to aerobic respiration. Combining this with the fact that the tricarboxylic acid (TCA) cycle was enriched for upregulated duplicated pairs, we map the categorized proteins onto the two pathways (Fig. 7). At least one of the proteins involved in each of the steps was upregulated, and in most cases the duplicated pairs were upregulated (i.e., *CIT1* and *CIT2*, *MDH1* and *MDH*3, and *ACS2* and *ACS1*). Interestingly, in cases where only one of a duplicated pair was within this pathway, we saw upregulation of just one of the proteins (i.e., *ACO1*, *LDP1*, or *KGD2*), namely, the one within the TCA cycle.

### Transcriptional divergence between duplicated genes plays different roles in WGDs and SSDs.

If TD of duplicated genes plays the same role in WGDs and SSDs, the same patterns should be observed. It has previously been noted that WGDs are more transcriptionally divergent than SSDs (30). Using our data, we find 910 WGDs to be TD in YPD at t_0_, compared to 824 SSDs, not a significant difference (Fisher’s exact test: *P* = 0.1389). However, when looking at the magnitude of the TD between duplicates, we observed a significant difference between the two types, with WGDs showing a higher TD than SSDs (Wilcoxon rank sum test: *P* = 0.01281) (Fig. 5B). It is worth noting that this difference of magnitude, between WGD and SSD, disappears if we look only at TD gene copies (Wilcoxon rank sum test: *P* = 0.1575).

To determine if the TD between the gene copies of WGDs and SSDs had different influences on the response to ethanol, we looked at how many TD duplicates were up- or downregulated in YPE compared to YPD at all three time points. For the duplicates *per se*, we had seen in the section above that upregulated genes contained more TD genes than expected (Fig. 4A). When separating out the two types of duplicates, it was seen that SSDs contained more TD upregulated genes than expected at all three time points (binomial test: t_0_, *P* = 2.399 × 10^−4^; t_100_, *P* = 7.239 × 10^−7^; t_110_, *P* = 3.622 × 10^−3^), as well as for downregulated genes at t_0_ and t_110_ (binomial test: t_0_, *P* = 0.02408; t_110_, *P* = 3.349 × 10^−3^). This is the opposite pattern from what we observed in WGDs, where neither up- nor downregulated genes, at any of the time points, had more TD genes than expected (Fig. 4A). To rule out that the limit set for a duplicated gene pair to be TD was not affecting our results, we redid the analysis for the TD limit going from equal expression to a 4-fold difference (Fig. S6). In general, the pattern did not change much as the TD limit was changed, in particular at the lower TD limits.

10.1128/mSystems.00416-20.6 FIG S6 Implication of transcriptional divergence limit on transcriptional response of duplicates. Shown is the influence of changing the TD limit, for which a duplicated pair is seen as being TD, on the enrichment analysis of responding duplicates which are TD. The analysis for the TD limit goes from equal expression to a fourfold difference. Download FIG S6, EPS file, 0.4 MB. Copyright © 2020 Sabater-Muñoz et al. 2020 Sabater-Muñoz et al. This content is distributed under the terms of the Creative Commons Attribution 4.0 International license.

**FIG 8:**
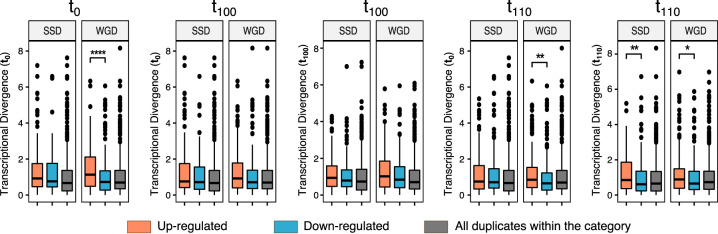
Distribution of transcriptional divergence per time point of the experimental evolution and per duplicate origin (SSDs and WGDs). A Wilcoxon rank test was performed to determine significant differences in the TD, indicated as *, **, ***, and ****, when the probabilities are *P* < 0.05, *P* < 0.005, and *P* < 10^−3^, and *P* < 10^−4^, respectively.

As we saw a difference between WGDs and SSDs with respect to the quantity of TD genes that reacted to the ethanol stress, we wanted to see if there was a difference with respect to the magnitude of the TD and ethanol response. First, we compared the TDs of up- and downregulated genes. The general pattern observed was that the WGDs show a statistical difference between the magnitudes of TD of up- and downregulated genes (Wilcoxon rank sum test: t_1_, *P* = 6.5 × 10^−5^; t_100_, *P* = 0.05; t_110_, *P *= 5.3 × 10^−3^) (Fig. 8). In contrast, SSD showed no difference between the magnitudes of the TD for up- and downregulated genes. Second, we wanted to see if the observed mean TD of differentially expressed genes was higher or lower than expected by chance. We compared the observed mean TD with the normal distribution build by random sampling of the same size from the corresponding pools (WGDs and SSDs). In this case, we observed similar patterns for both WGDs and SSDs. The TD of upregulated genes exhibits the expected mean of TD. One interesting thing for both was that the mean TD at t_110_ was significant only when calculated from t_110_ but not from t_0_, indicating that the transcriptional background has an influence on the response of the duplicated genes in ethanol stress. Furthermore, neither WGDs nor SSDs showed a significantly higher mean of TD of downregulated genes than expected, at all time points (Fig. 4B).

## DISCUSSION

### Large transcriptional response to ethanol stress.

One of the central mechanisms of unicellular organisms, and particularly nonmobile ones, is sensing and responding to changes in the environment. This is especially essential when the organism endures stress. Here, we show how changing the carbon source from glucose to ethanol leads to large transcriptional changes in the yeast S. cerevisiae. These changes are observed in a large percentage of the S. cerevisiae genes and encode a wide range of functions. Such genome-wide transcriptional changes have been shown before to take place in the response to numerous stresses, including glucose restriction (31), glycerol as the only carbon source (19), oxidative stress (32–34), environmental estrogen (35), acid tolerance (36, 37), and thermal resilience (38), among others (39). Indeed, when the initial population was switched from a glucose-containing medium to one that contained only ethanol as carbon source, hundreds of genes altered their expression. With the medium not containing glucose, the yeast is performing aerobic respiration (4, 5, 7), and the presence of ethanol induces oxidative stress of the cells (40–45), which is reflected in our results, where we have an enrichment of “oxidation-reduction process” and “aerobic respiration” gene terms in the upregulated gene classes. Hence, two clear transcriptional patterns were observed during exposure to ethanol: (i) an upregulation of genes involved in stress response and (ii) a downregulation of ribosomal biogenesis or energy-dependent processes. Our observations agree with the suggested tradeoff cellular response (transcriptional rewiring of central metabolism) to environmental stress on yeast growth rate (36, 39, 46, 47).

### The genetic background influences the transcriptional response to ethanol.

The genetic background of a population influences its capability to grow and respond to stress (48–50). In this study, evolving a population of S. cerevisiae for a large number of generations had huge influences on the transcriptional response to ethanol, as well as increasing the growth rate in the evolved medium (YPD). We observed transcriptional changes between the evolved and the nonevolved ancestral population. This change is likely due to a genetic change in the population, as the original ancestral population had low variability as it originated from a single clone and the evolved population gained genetic variability through the evolution experiment, as described recently (51). An interesting result from our study is that the evolved population improved its fitness in the evolved medium (YPD) but showed no change in the fitness when grown in ethanol (YPE). It has previously been shown that diversification can lead to exaptation in nonevolved environments (19, 33, 51, 52). There are multiple possible reasons for us not observing this in our populations. First, increasing fitness to ethanol is hard. Many of the studies which have observed increased tolerance to ethanol see an increase of the ploidy of chromosome III (53, 54); this occurs only in diploid and polyploid S. cerevisiae, and we are evolving a haploid population. Second, our ancestral population might have been at a local maximum in the ethanol fitness landscape of the population. Last, despite evolving our populations for approximately 660 generations, it might not have been long enough to acquire any exaptation to ethanol, deserving further study of the mutational landscape of these populations.

### Going from acute to chronic exposure of ethanol rewires the transcriptome.

The evolution of the yeast population in the presence of ethanol (the t_110_ populations) uncovered the regulatory changes that occur as the population reacts to acute and chronic exposure. It has previously been suggested that reducing the growth rate can lead to increased stress tolerance by redirecting the resources (47). The chronic-exposure population (evolved population in ethanol, YPE_t_110_) showed an enrichment of upregulation of genes involved in oxidative stress, so overall these populations were upregulating more genes involved in stress response, an indication of higher allocation of resources to stress tolerance. This agrees with the fact that we observed a lower growth rate on ethanol for the evolved population in ethanol than for the population that evolved in YPD.

### Duplicated genes play an important role in the response to ethanol.

The first response, of an organism to stress, is though regulatory reprogramming; hence, plasticity of the transcriptome will determine the potential for adapting to a new environment (55–57). However, this link is still not fully understood, enthralling scientists for the past 40 years and becoming of great importance recently (58), and that is predominately down to the difficulty of mapping phenotypes to genotype and assigning transcriptional changes to phenotypic variations. In this work, we clearly see a link between transcriptional variations, phenotypic response to ethanol, and gene copy number (referring here to duplicates), as the transcriptionally altered genes are enriched for duplicated genes. The classical theory behind the evolution of duplicated genes states that one gene copy is able to evolve without or with reduced selection constraints as the other gene copy is performing the ancestral function (28, 59, 60). Diversification of the gene copies happens not only at the functional level but also at the expression level (34, 61, 62). The diversification at the expression level could open up the possibility to diverge functionally, as the rate of evolution is highly linked to its expression, although recently it has been shown that low-expression transcription factors adapt through cooperation rather than functional divergence (34, 63). It has been suggested that the whole-genome duplication even facilitated the *Saccharomyces* yeast to evolve the ability to ferment sugars under anaerobic conditions, which is not the case for other yeasts (reviewed in references 64 to 66). Here, we are forcing the yeast to use ethanol as the sole carbon source, meaning it has to perform respiration instead for fermentation, not requiring the Crabtree effect-implicated genes. In correlation with this, we observe that most of the changes of the duplicated genes were downregulation of WGD, consistent with the hypothesis that WGDs were providing the raw material for conservation of dosage-sensitive genes involved in both rewiring of rapid growth elements (ribosomal protein genes) and divergent regulation and specialization of gluconeogenesis-ethanol consumption phase versus glycolysis-ethanol production (8, 65). Taken as a whole, the rewiring of the transcriptome, and in particular the duplicated genes, indicates that the yeast cell goes into energy preservation when the carbon source is switched to ethanol.

### The transcriptional background and the response to ethanol.

In plants, it has been observed that duplicated genes diverge transcriptionally soon after duplication (66–69). Furthermore, a correlation between the divergence from the ancestral expression level and stress response has also been observed in plants (70). These all indicate that duplication and expression divergence are linked to adaptation and stress response (67, 71). In yeast, duplicated genes have also been shown to be transcriptionally diverged, particularly in WGD (19). In a wider study looking at transcriptional changes of duplicated genes under different stress conditions, it was observed that one of the gene copies was more transcriptionally plastic than the other (23). These all indicate that transcriptional divergence plays an important role in maintaining duplicated genes in the genome and expanding the phenotypic plasticity of the organism. Here, we observe that transcriptional divergence between gene copies is correlated with response to ethanol. In particular, responding duplicates have higher transcriptional divergence than expected. However, WGD and SSD have different parameters by which the TD influences the response to ethanol. The magnitude of the TD is important for WGD, where in contrast the number of genes with TD is important for SSD. One interesting thing that we observed in this study is the change of TD of the duplicated genes throughout our experiment and that this change was correlated with the response to ethanol, indicating that the transcriptional background is important for the actual stress response and this can change relatively quickly.

### Concluding remarks.

The recent advances in next-generation sequencing technologies coupled with the decrease of their prices have increased general interest in determining the role of polyploidy and transcriptional plasticity in ecological shifts or lifestyles. The switch to use ethanol as sole carbon source implied a yeast cell reprogramming to energy preservation with low growth rate but with similar biomass production due to transcriptional reprogramming of duplicates, especially those of the TCA cycle. In this work, we have unveiled that TD between duplicates and the transcriptional background affect duplicates’ response to ethanol, with the magnitude of the TD being especially important for WGDs.

## MATERIALS AND METHODS

### Yeast culture and experimental evolution.

The Saccharomyces cerevisiae strain Y06240 (BY4741: *MAT***a**; *his3D1*; *leud2D0*; *met15D0*; *ura3D0*; *msh2*::*kanMX4*) was used as described previously (22, 72). Briefly, a homogeneous population founded by growing a colony in a liquid culture of rich medium (YPD: 2% [wt/vol] Bacto peptone, 1% [wt/vol] yeast extract, 2% [wt/vol] dextrose; supplemented with 100 μg/ml kanamycin) (t_0_) was evolved through daily bottlenecks (1%) for 100 days (t_100_; ∼660 generations), in 5 ml of YPD medium in 50-ml Corning tubes, at 28°C and 220 rpm. From passage 100 (t_100_), the population a1 was divided into two sublines, each with three biological replicates. One subline was grown in YPD medium as control (lines Da1), whereas the second subline (lines Ea1) was grown in a medium containing 3% ethanol as the sole carbon source (YPE: 3% [vol/vol] ethanol, 2% [wt/vol] Bacto peptone, 1% [wt/vol] yeast extract; supplemented with 100 μg/ml kanamycin). The populations were evolved for another 10 passages, with a daily bottleneck of 10% of population, in 5 ml of the corresponding medium, as indicated previously. Each 10 passages, a fossil record of each line was established by preserving the entire population in 25% glycerol solution at −80°C (Fig. 1).

### Growth characterization.

Growth parameters for t_0_, t_100_, and t_110_ were obtained using the Bioscreen C plate-reader system (Oy Growth Curves Ab Ltd., Helsinki, Finland) as described in reference 72. Briefly, each time point was precultured overnight at 28°C, from the corresponding fossil record, and used to inoculate 200 μl of fresh medium (YPD and/or YPE) to an initial optical density at 600 nm (OD_600_) of 0.06 to 0.07, distributed in 100-well honeycomb plates, with 6 to 7 technical replicates. The experiment was run for 78 h at 28°C with continuous shaking (high level) and taking OD_600_ measurements (brown filter) every 15 min. Each run contained at least 3 controls for each medium (uninoculated fresh medium). The data were analyzed with Growthcurver v.0.3.0 under R-studio (73).

### RNA extraction and transcriptomic analysis.

The RNA profiling was performed at the t_0_, t_100_, and t_110_ time points as indicated in Fig. 1A, following the same procedures as previously used (22, 72). rRNA-depleted RNA (Illumina) libraries were constructed and sequenced at the Genomic Core Facility at Servicio Central de Soporte a la Investigacion Experimental (SCSIE) from the University of Valencia, Spain. Reads (trimmed) were aligned with Bowtie2 (up to two mismatches accepted) to the reference S288c strain genome (only coding sequences [CDS]). Statistical assessment of differential gene expression was done with edgeR (74), setting false-discovery rate (FDR) at <0.005, and applying BY correction for *P* value (0.005).

### Identification of duplicated genes, functional classification, and visualization.

Paralogous pairs of duplicated genes were divided into two groups according to their origin mechanism: whole-genome duplicates (WGDs) or small-scale duplicates (SSDs). WGDs (555 pairs) were extracted from the reconciled YGOB list (Yeast Gene Order Browser, last accessed March 2018; http://wolfe.gen.tcd.ie//ygob [75]). SSDs (560 pairs) were identified after best reciprocal hits from all-against-all BLAST searches using BLASTP with an E value cutoff of 1E−5 and a 50-bit score (76), selecting only those that exhibit a distribution of synonymous substitutions similar to WGDs (3, 26). Differential expressed genes were further classified according to their gene ontology (GO) term as implemented in the R package clusterProfiler (77), followed by an enrichment analysis with a *P* value cutoff of <0.01 and with the *P* value being adjusted with the Benjamini and Hochberg (78) method.

### Software.

Unless otherwise indicated, statistics were performed using the appropriate packages in R v 3.5.1 (R Core Team [2018]).

### Data availability.

Raw reads are available from the Sequence Read Archive (SRA) with accession numbers PRJNA321113 (t_0_ in YPD and YPE), PRJNA610243 (a1t_100_ in YPD), PRJNA610541 (a1t_100_ in YPE), PRJNA610474 (Da1t_110_ in YPD), and PRJNA610515 (Ea1t_110_ in YPE).
